# Promoting xenomorphic patient-facing AIs: The case against anthropomorphism in medical AIs

**DOI:** 10.1038/s41746-025-02046-7

**Published:** 2025-11-17

**Authors:** Stephen R. Milford, Emma Herger, Johanna Eichinger, Bernice S. Elger

**Affiliations:** 1 Institute for Biomedical Ethics, Basel University, Basel, Switzerland; 2 North-West University, Potchefstroom, South Africa; 3 University Hospital Basel, University Psychiatric Clinics Basel, Geriatric University Hospital FELIX PLATTER Basel, University Children’s Hospital Basel, Basel, Switzerland; 4 Institute of Midwifery and Reproductive Health, Zurich University of Applied Sciences, Zurich, Switzerland; 5 Center for Legal Medicine, University of Geneva, Geneva, Switzerland

**Keywords:** Medical ethics, Health policy

## Abstract

The rapid emergence of patient-facing medical artificial intelligence (MAI) raises pressing questions about its design and impact on healthcare. Current anthropomorphic design strategies, which endow AIs with human-like features, are based on a reductivist understanding of healthcare and risks undermining the integrity of doctor–patient relationships (DPRs) that are foundational to positive health outcomes. This paper argues for a xenomorphic approach—designing MAIs with decidedly non-human, even alien-like characteristics. By distinguishing AIs from human physicians as far as possible, xenomorphic design may avoid direct competition with human relational roles, protecting the ontological distinctiveness of DPRs. Importantly, we suggest that such designs may foster a new class of therapeutic relationships with patients: non-human but nonetheless health-affirming. Analogous to the established benefits of animal-assisted therapies, xenomorphic MAIs could provide complementary sources of trust, comfort, and support without supplanting essential human care. The xenomorphic approach may therefore not only avoid the conceptual and practical challenges of anthropomorphism but also offer an innovative path to expand therapeutic possibilities and improve health outcomes.

## Introduction

Over the last few decades, AI has begun to revolutionise healthcare. In the past, medical AI (MAI) applications have been confined to what may be described as the backend of healthcare—systems of healthcare not directly interacting with patients. Recently, however, this has begun to change. With new developments in natural language processing (NLP) based on large language models (LLM) deployed in the form of medical chatbots, patients themselves are beginning to directly interact with MAI chatbots like Emma or Vivibot^1,2^. In fact, more than 120 patient-facing medical AIs are currently in use, or in the process of being deployed^3^.

In many ways, this is good news. While results are mixed, there are indications that in certain circumstances chatbots have demonstrated diagnostic accuracy akin to human doctors^4–6^. This is true not only for purpose-built MAIs such as Ada, Buoy or Babylon, but also for free, publicly accessible chatbots. ChatGPT, for example, has passed numerous state medical licensing exams, from all three of the US licencing exams to those in Germany and Italy^6–9^. Indeed, purpose-built, public-facing MAI chatbots have already been deployed in state healthcare systems such as the NHS^10^.

While we do not envision that MAI could fully replace a human doctor, there are benefits to patient-facing MAI, particularly in contexts where access to human doctors and affordable healthcare is restricted, as long as this does not diminish our efforts to ensure equitable access to human doctors. Yet, as the preceding sections of this paper will demonstrate, it must be borne in mind that healthcare is more than simply alleviating physical or mental ailments. Diagnostic accuracy will not automatically lead to better health outcomes^11^. There is ample evidence to demonstrate that doctor-patient relationships (DPRs) have causal links to health outcomes^12–15^. Health care is a decidedly relational endeavour.

Consequently, the rise and widespread use of patient-facing MAI raises numerous practical and ethical challenges around how best patients can relate in health-affirming ways to MAI. This paper will not engage exhaustively in these numerous challenges. Rather, it will focus on the crucial aspect of the manner in which we could, or should, design the appearance of new MAIs. With this in mind, our paper will explore the recent drive to anthropomorphise AI generally, the normative implications of the relational aspects of health care, and challenge the anthropomorphic drive behind patient-facing MAI. Instead of advocating for a highly competent human-like AI physician, such as the Emergency Medical Hologram of the sci-fi series Star Trek: Voyager (1995–2001) played by Robert Picardo, we will argue for an entirely non-anthropomorphic approach: a xenomorphic approach. By ‘anthropomorphic’, we refer to the approach taken by researchers when designing AIs with human-like characteristics to encourage members of the public to perceive AIs as more human. In comparison, the ‘xenomorphic’ approach seeks to design AIs with decidedly non-humanlike characteristics, almost alien, so as to distinguish AIs from human beings as far as possible.

To demonstrate the legitimacy of this approach, we will begin our discussions with a brief overview of the current tendency to anthropomorphise AIs generally. We will argue that while great strides have been made to identify the kinds of humanlike appearances that engender trust, applying these to modern AIs results in a paradox known as the ‘uncanny valley’ that appears—at least for now—to be insurmountable. Rather than identify ways to circumvent this paradox, our next section will argue against the current anthropomorphic tendency, especially in medicine. We will demonstrate that human medicine is a decidedly humanly relational endeavour and that human DRP cannot and should not be substituted by non-human relationships. Notwithstanding this, our third section will advocate for the value of non-human relationships that promote positive health outcomes. There, we will argue for the benefit of creating patient-facing AIs with decidedly alienlike characteristics so as to protect current health-affirming human DPRs while still promoting additional non-human health-affirming relationships. The paper will end with a brief discussion of the advantages and limitations of the xenomorphic approach.

## Anthropomorphising AI

As public engagement with AI increases, much research has been conducted as to how AIs can be presented to the public to engender trust. This appears, *prima facia*, achievable. Humans have an innate ability to assign human-like minds to the world around us. We routinely attribute humanlike mental capacities to objects and beings through what Epley describes as the ‘very ordinary process of social cognition underlying anthropomorphism’^16^. This extends to innate objects such as cars or cell phones, living beings such as animals, but also to AIs generally. We use personal pronouns, or even human-like names, to speak of mindless objects such as ships and planes, while pets are regularly treated as ‘part of the family.’ AI is no exception.

It is well established that humans perceive AIs as having some form of personality, and consequently, they instinctively respond to them in human-like ways, such as being polite when asking AIs questions^17–19^. This is particularly true for AIs with human-like features^20–22^. Consequently, developers have gone to great lengths to attempt to further anthropomorphise AI and present it in human-like ways to prompt human-AI trust^23^. This is particularly true in the case of medical AI, where doctor-patient relationships play a key role^24,25^. Here, researchers have sought to capitalise on empirical data regarding the physical appearances of human beings that most engender trust in human-to-human relationships with a naive belief that this may be extended not only to social robots, but patient-facing medical AIs as well. That is to say, researchers believe that if we can identify the exact physical features that engender trust in human-to-human relationships and mimic these in medical AIs such as patient-facing medical chatbots, we could establish an AI doctor–patient relationship that is similar (or even superior) to a human doctor-patient relationship.

There exists extensive research into which human features most influence an observer’s perception of trust. This includes facial features such as skin shade (with darker shades associated with more trustworthiness); brow ridge (with higher brow ridges associated with more trustworthiness); the weight of a face (light faces being associated with higher levels of trustworthiness); forehead (taller and smaller foreheads being more trustworthy, this was particularly true among female faces^20^); as well as the face’s brow–nose–chin ratio (with smaller ratios being associated with higher levels of trust^22^). The shape, movement, and colour of eyes are particularly important^26^, with some evidence suggesting that brown eyes are perceived as more trustworthy^27^. Beyond facial features, the general perception of the face has been shown to either engender trust or mistrust. For example, smiling intensely has a positive effect on perceived trustworthiness^28^, as does the observer’s perception of whether or not the face is happy or angry^29^.

Furthermore, research has demonstrated the impact of certain ethnicities, genders, ages, attire, and general perceived attractiveness on trustworthiness. Here, Asian and female faces (being generally perceived as having shallower chins, higher brow-ridges, and longer nose bridges) are perceived as highly trustworthy^20,29^. Perceived attractiveness and professional attire (such as wearing white coats) engendered more trust than those who were perceived as unattractive, or casual^22,28,30,31^.

Interestingly, in the context of age, results have been mixed. Some have demonstrated that a higher rank (e.g. professor) was consistently associated with higher levels of trustworthiness, and yet older physicians were associated with lower levels of trust^32^, yet other research has demonstrated the opposite, with older surgeons associated with higher levels of trust^33^.

To all this could be added the concepts of familiarity, typicality, and concordance or discordance. Research is mixed in this regard^34,35^ cf^36^. It has been shown that age/gender/racial concordance leads to higher trust scores among research participants. That is to say, when the age/gender/race of the physician is in concordance with a patient, higher levels of trust are afforded to the physician^32,37,38^. There are nuances to this that must be considered. For example, Rogo-Gupta et al. demonstrated that gender-concordant physician-doctor pairs received significantly lower trustworthy scores than discordant pairs^37^, while Schmid et al. demonstrated that when a patient was part of the ethnic majority, concordance was associated with higher trustworthiness, while the opposite was true for ethnic minority populations^26^.

At this point, a very brief interjection should be noted. While there exists research into specific appearance features that engender trust in human-to-human relationships, it should be noted that much of this research is culturally insensitive and therefore non-generalisable. It is arguable that trust emerges within cultural and social contexts. Much of the research on this topic ignores this critical context. Furthermore, insofar as this research is often undertaken by dominant cultures (often in the West), it is arguable that it may even be perceived as colonialist, propagating the views of dominant cultures on who is and who is not trustworthy. It is fair to say that such approaches encourage bias of many kinds: gender, cultural, sexual, etc. Here, the authors are not affirming the suitability of the research undertaken for the task of designing AIs; we are merely describing the approach others may take. We will very shortly come to critique this approach.

**Fig. 1:**
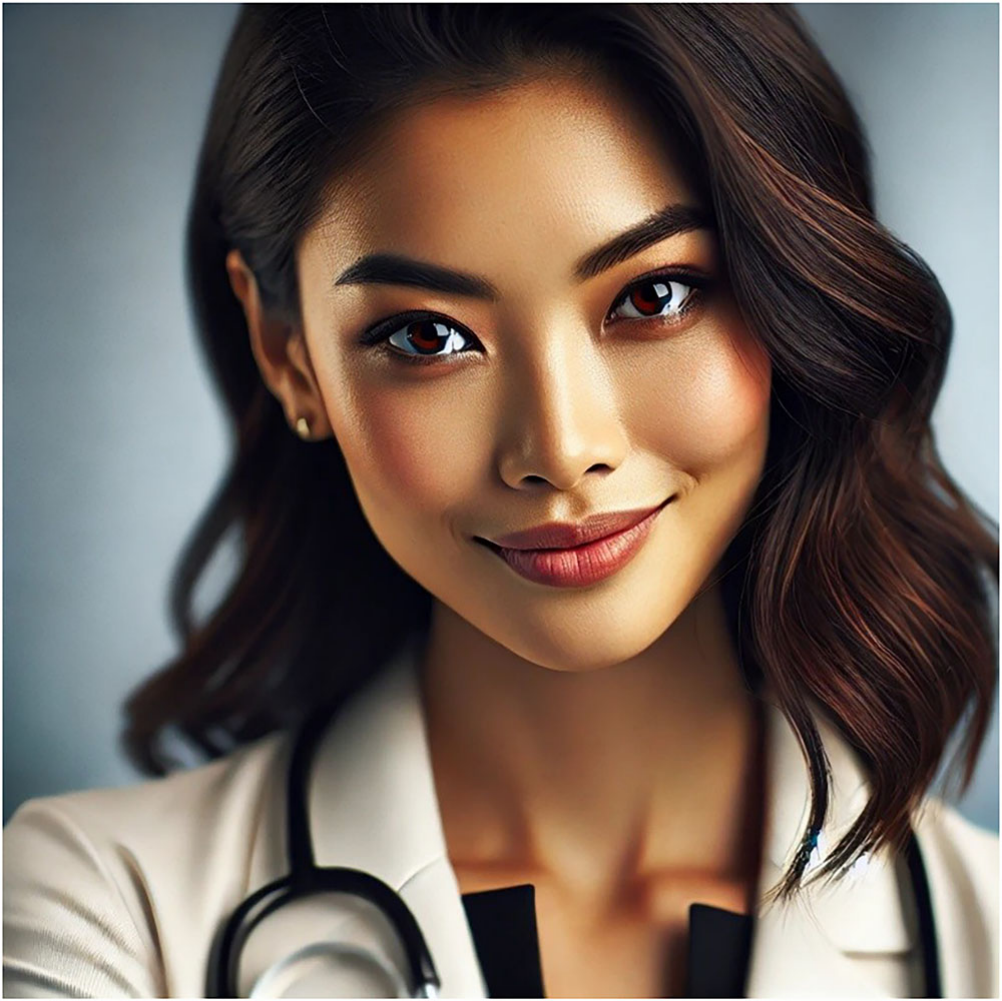
An MAI appearance based on well-established trust-engendering features as depicted by ChatGPT. Prompt: “Please produce a picture of an attractive, well-dressed, brown eyed, slightly darker-skinned, light-weight, Asian female face with a high brow-ridge, tall, small forehead, a small brow–nose–chin ratio, who smiles intensely and is called Prof. Dr Medical AI.” 2.3.2025.

Considering the complexity of the research outlined above, and setting aside our interjection, it may be possible for one to argue for three options as to how medical chatbot AIs should be presented to patients. Each option presents advantages and challenges. (1) One could argue that a single, or perhaps a limited set of universal MAI appearances should be presented to all patients everywhere. That is to say, MAIs should be developed to display fixed features that do not change depending on the patient and context. Based on some of the research outlined above, one could argue that the type of features that engender greatest trust might incorporate an attractive, well-dressed, brown eyed, slightly darker-shaded, light-weight, Asian female face with a high brow-ridge, tall, small forehead, a small brow–nose–chin ratio, who smiles intensely and is called Prof. Dr. Medical AI (Fig. 1). The research indicates that this appearance is more likely to engender trust, build better AI-human relationships and consequently more positive health outcomes. Apart from being highly insensitive to different cultures and social contexts (thereby promoting bias and undermining the very trust it would seek to engender), this approach—which provides patients with no choice—seems almost undemocratic and counter to the principle of autonomy.

(2) One could argue that MAIs should automatically adjust their appearances depending on the patients they are faced with and the principles of concordance and familiarity according to age, gender, and culture. For example, a 50-year-old ethnic majority patient should be presented with an MAI face that is likewise majority ethnic and approximately 50 years old. In the case of an ethnic minority patient, an ethnic majority MAI. This approach has the advantage of incorporating the research into patient-doctor concordance and trust, thus being potentially better for a wider range of patients than option 1. However, automatically modifying the AI according to patients, without their agreement, may be perceived as manipulative and, therefore, could backfire in its intended goal of engendering trust.

(3) In light of the principle of autonomy and modern personalised medicine, one may argue that we should let patients themselves decide what appearance they would like their AI doctor to have. Such an approach has been suggested in terms of the linguistic style of chatbot medical AIs^39^. Some, for example, might prefer a female AI of a certain ethnicity who wears certain attire than a middle-aged balding male MAI. Patients could choose gender, culture, or even age-neutral MAIs. This would certainly promote the democratisation of healthcare and the autonomy of patients. However, how one ensures that the MAIs appearance does indeed lead to better health outcomes, rather than just conforming to the wishes of patients, would be a challenge. There are questions about how much patients would actually follow the medical advice of an MAI if they chose to dress it up as a clown. Worse still, one would be hard-pressed to argue that a concordant MAI who is obese and perhaps smokes during a consultation with a smoker suffering from type 2 diabetes and lung cancer would encourage the kind of lifestyle behaviours that might foster positive health outcomes.

**Fig. 2:**
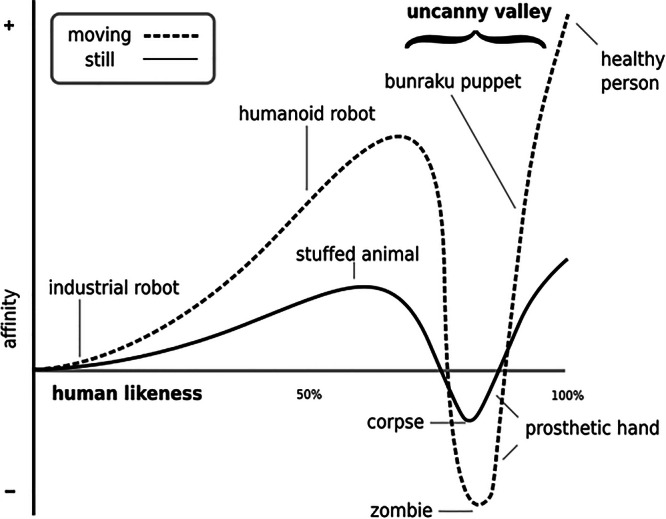
The uncanny valley as depicted by Mori et al.^40^. This graph depicts the phenomenon whereby human affinity to a robot initially increases the more humanlike the robot appears, until the robot appears very humanlike, at which point human affinity quickly reduces.

Beyond the challenges laid out in each of the options above is the more significant problem of what has come to be known as the uncanny valley. First introduced in 1970 by Masahiro Mori^40,41^, the uncanny valley speaks to an interesting paradox. Increasing a robot’s human likeness initially elicits more positive and empathetic responses from observers; however, once the robot becomes almost human, responses abruptly turn to strong revulsion. When plotted on a graph, the reactions are indicated by a steep decrease in acceptance where the appearance is only slightly human, to a steep increase, the closer to human-like the robot appears, followed by a subsequent steep decrease at the point that the robot becomes almost human-like. Hence the term, ‘uncanny valley’—referring to the trough just before the AIs appearance is uncannily human (see Fig. 2)^40^. According to this principle, AIs with almost human-looking appearances seem overly ‘strange’, perhaps even “unnatural” to some observers and produce feelings of disgust, resulting in reduced empathetic responses required for AI–human interaction.

This is particularly true when both human and non-human elements are mixed. For example, if a robot has a synthetic voice, or if its animation is less human-like than its voice or cognitive abilities. It is arguable that where one or more of its abilities are advanced, and do not match other features, a mismatch in expectation causes uncertainty in observers. For example, highly human-like appearances can lead to expectations that certain behaviours are present (such as fluid motion or speech). This may result in a ‘prediction error’ within the neurology of human observers who base their predictions on their expectations. There is a suggestion that as uncanny AIs become commonplace, human perceptual systems will re-tune^42,43^. While this may be the case, there is reason to argue for the avoidance of humanlike MAIs altogether, especially when one considers the fundamental nature of medicine itself.

## The relational nature of medicine

The link between DPRs and health outcomes has been well established^12,14,44^. That is to say, the relationships patients have with their physicians have a causal link to their health outcomes. Good relationships are associated with better health outcomes and vice versa. Taking this link seriously may lead us to argue that anthropomorphising MAI is desirable. As our discussions above have indicated, there are appearance attributes that garner trust in human relationships. If we can identify which of the appearance attributes of human physicians garner health-affirming DPRs, we may be able to incorporate these appearances into patient-facing MAIs. Theoretically, this should lead to better MAI-patient relationships and consequently better health outcomes if we can overcome the uncanny valley paradox. Should this be the case, it would be arguable that we would have a moral imperative to design patient-facing MAIs with these appearance attributes according to the three options we laid out in the previous section. Yet, even if this were possible, there are significant reasons why we should avoid the approach of anthropomorphising MAI.

The approach to anthropomorphise MAI by providing it with a human-like appearance is based on a positivist, reductivist paradigm adopted from the scientific methodology^45^. That is to say, it is based on the idea that one can distil the features of human-to-human relationships that promote health outcomes and mechanistically apply these to non-human entities. It is understandable that many may wish to use a reductionist or positivist approach to developing MAI, considering that many of the major breakthroughs in healthcare have come from these approaches. As a consequence, some healthcare professionals (HCPs) have been ‘enculturated’^45^ into this paradigm. For these professionals, measures of healthcare success are often reported as statistical data on attributes such as recovery or survival. Indeed, many discussions on the efficacy of DPRs are presented in these terms, relating DPRs to health outcomes as measures of recovery, patient adherence, or even improving health-related behaviours such as ceasing smoking, decreasing drinking and stress; increasing sleep or exercise^14^. These measures present the healthcare context as one focused entirely on the physical alleviation/management of illness, disease, or injury. Seen from this perspective, designing AIs to be effective at fostering DPRs that are effective at eliciting certain human responses—such as treatment adherence or health-related behavioural changes—appears legitimate.

However, this reductive paradigm is not suited to the broader context of human health. Good healthcare is more than simply the alleviation of physical ailments. Pellegrino, for example, urges HCPs to develop a sound philosophical basis for healthcare that can provide effective normative principles^46–48^. He has demonstrated normatively that the positivistic, reductivist philosophy of healthcare can blind us to important aspects of what it means to be healthy. This obsession can take our focus away from the lived experience of illness and prevent us from seeing what is often most important to our patients. This is particularly pertinent to contexts where medical interventions become futile. In such contexts, such as in palliative care, we need to broaden our understanding of what *healing* and *health* really mean for patients who will never physically recover.

Likewise, Mount demonstrates that healthcare can be defined as ‘a relational process involving movement[s] towards an experience of integrity and wholeness’^45^. Here, Mount (the first physician to create a palliative care unit in North America) argues that the healthcare context requires a robust understanding of medicine’s mandate to promote a notion of healing as caring for the whole person and not merely their physical ailment. He, along with others, understands illness as more than a physical assault on the body. Rather, it is an assault on one’s own sense of integrity, one’s sense of being. Illness leads to a fundamental sense of disintegration, loss of control, even alienation from oneself^45^, almost as if one is cast adrift in a foreign land^49^. Being healed is marked by the experience of wholeness and personal integrity that is encapsulated by the notion of human flourishing^50^. In this view, HCPs are not scientists experimenting on the physical world. They are not ‘morally neutral technicians’^45^ but moral agents. They are active participants in the restoration of human wholeness brought on by the assault of illness. They can only be active participants in that they (as human beings) are themselves seeking this wholeness and have themselves experienced the disintegration of human flourishing brought on by illness or death.

From this normative understanding promoted by physicians such as Pellegrino and Mount comes an understanding of human health that goes beyond a narrow focus on the physical well-being of our patients. Human healthcare is a uniquely human relational experience and a moral enterprise^47,48^. It is uniquely human because it is aimed at the restoration of human wholeness by human beings. HCPs seek to aid their human patients to experience human flourishing once again in the face of an assault on their very beings. It is humanly relational in that human physicians (who themselves are seeking their own human flourishing) pursue the flourishing of their human patients in the context of the human community. Here, the physician is more than simply a machine, a means to an end; they are a partner in the very experience of human flourishing and wholeness.

This flourishing and wholeness cannot be achieved without the experience of inter-human relationships. One can make this claim partly as a result of an understanding of the ontological nature of human being itself that must, by definition, include personal relationships with other human beings (in order to be human) in significant life moments, often contextualised by healthcare (e.g., birth, illness, and death). Space prohibits an extended philosophical discussion on the definition and ontology of *humanity*. Many normative frameworks can be used to justify this claim. For example, philosophical relational constructions of humanity, such as those expounded on by MacIntyre^51^, or Taylor^52^; the phenomenological anthropology of Levinas^53^, Heidegger^54^, or Buber^55^; recent anthropology and sociology theories expounded on by sociologists such as Mead^56^, or Mbiti^57^; or even theological anthropological claims by theologians such as Zizioulas^58^ or Shults^59^. There is widespread agreement that human beings are dialogically formed as human beings by their interactions with other human beings in critical moments such as birth, illness, and death.

Consequently, our understanding of DPRs (based on a human-to-human relational ontological) will determine what we regard as the obligations each partner in the relationship owes each other^46^. If we understand DPRs as merely the conduit for the alleviation of physical ailments, we might seek to design MAIs through a reductivist approach: seeking those aspects of the doctor that lead to better health outcomes. If, on the other hand, we understand healthcare, and with it the DPR, as a uniquely human experience that brings with it the moral obligation to promote human integrity, wholeness, and flourishing, designing human-like patient-facing MAIs might exacerbate the problem of the uncanny valley. The more we try to create human-like AIs, the more we may find them repulsive to patients. Not merely because they may never be entirely human-like, but because they simply cannot be human and, as such, cannot substitute for a human being in the context of deeply intimate human relationships. Introducing these uncannily human-like doctors into the lived experience of illness—an experience whereby patients seek out human contact to restore human wholeness and flourishing—would be futile. It may even be destructive if it were to undermine current human DPRs.

Thus, an entirely different approach is needed.

## An Alien doctor

Recognising the distinctly human nature of medicine begs the questions: What role, if any, can patient-facing MAI play? Does it even make sense to speak about DPRs in the context of MAIs if MAIs cannot substitute for human doctors? While it is true that human doctors, as humans, will always play a distinctive role within the context of human medicine, this is not to say that other relationships may not be helpful nor contribute to health outcomes, albeit in decidedly different roles. Indeed, patients must often relate to non-human healthcare actors in both intimate and non-intimate ways. For example, patients relate to private and public institutions (e.g., hospitals or clinics). These relationships, while non-intimate, are significant for health outcomes, and a great deal of work has been done to improve the institutions themselves to engender trust and relate to patients in health-affirming ways. For example, improving workflows, administration, layout, disability access, etc. These relationships may be described as non-intimate relations in that they are between patients and institutions/organisations, yet they still have direct bearings on health outcomes.

Could it be that MAI is simply another tool within this context, like an X-ray machine or an echograph? Is it possible that MAI might be deployed (and therefore accordingly designed) solely as a supplement to human HCPs and never a substitute? While some may argue that MAI is just another medical tool, there is ample reason to believe that MAI is exceptional in that it goes beyond merely being an aid for a physician to providing recommendations and treatment plans^60–62^. Indeed, the very discussions about anthropomorphising AI, and in particular the personalising manner with which human beings interact with AI, lead us to believe that, overall, the public sees new AI technologies such as AI chatbots as more than simply tools. That is to say, there is evidence that the public is not only capable of, but desires intimate relationships with AIs generally, and in particular, MAI. Duffy^63^ has long ago argued that, as AIs enter our social space, humans will project their interpretations of AI actions in much the same way as they do for pets. This, to use Epley’s phrasing, is ‘exceptionally ordinary’^16^. It is unrealistic, therefore, to contend that AIs will be used purely and solely as supplements to DPRs. It is widely accepted that MAI should never substitute for a human doctor, and yet there is already evidence to suggest that even non-anthropomorphic, non-medical AIs such as ChatGPT are being used as substitutes for medical doctors^64–66^. How much more so, should AI look, sound, and behave as a human being?

Rather than continuing a reductivist approach to designing patient-facing AIs to mimic human doctors’ appearances, it is argued here that we take inspiration from Coeckelbergh’s robot ethics^67^ and move toward a more experiential/interactive experience of interacting with personal AIs that radically distinguishes MAIs from human doctors. MAIs could be designed in such a way as to play a non-human intimate relational role that promotes positive health outcomes without commingling or conflating these with the experience of human doctors, thereby jettisoning the anthropomorphising approach to designing MAIs in favour of a xenomorphic approach. By presenting MAIs as an entirely new category of being to which patients can relate in intimate ways, we may protect current human DPRs while making space for additional non-human relationships that can further promote positive health outcomes. Rather than designing MAIs to look like human doctors with human-like facial features and attire, MAIs should be designed to be entirely alien-like. By this, we do not mean, as Mori argues^68^, that MAI should be presented as abstract forms, such as moving streams of light on a computer screen. There is evidence to suggest that humans interact best with embodied beings^69–71^.

Our argument is that MAIs could be presented as embodied (physical or virtual) but in entirely alien-looking ways. MAIs could, for example, present with abnormally large heads, four arms, no nose, three eyes and scaly skin. They could speak in fluid ways, but using distinctly non-human-like voices. While at first the public might find this strange, amusing even, it is here hypothesised that in time the public may come to accept these alien-looking MAIs as simply another non-human being with which we must share reality; just as we share reality with strange looking animals, animals that may have eight legs, feathers, beaks where mouths should be, or voices that sound like songs under water.

Before this seemingly radical idea is completely rejected as science fiction, consider the existing quotidian non-human intimate relationships that are already part of the healthcare context. For example, therapy animals. Who would have thought that it might be helpful to bring a dog into a hospital for therapeutic purposes, much less horses^72^, pigs, alpacas and even dolphins^73^. Patients are known to form deep, intimate bonds with these ‘strange’ beings that directly impact their health outcomes. This extends to emotional support animals, or guide dogs that assist visually impaired people to manage their impairments.

If human beings have been shown to have meaningful, intimate, health-affirming relationships with such strange-looking non-human beings, how much more might they benefit from a non-human-looking advanced intelligence whose very existence is based on, and expertise lies in, providing medical assistance? Indeed, we are not the first to argue that AI should not be understood in human terms but rather in animal terms. Darling has long argued that we should think of AIs not as humans but as animals^74–76^. Just as we have harnessed the power of animals to aid us in war and work, so too, argues Darling, we should harness AI to support rather than replace human skills and abilities.

Some have argued that such ‘ersatzcompanions’ are ethically problematic in that they cannot truly form emotional bonds with humans as animals can. For authors such as Sparrow, AIs cannot truly form ‘real’ relationships with humans in the same way animals may^77^. It is, therefore, ethically problematic to suppose (or even encourage) the allegory of AI as animal-like beings. However, unlike Darling and others, we do not feel the need to de-emotionalise nor de-personalise human-AI relationships^75^. While we agree that the anthropomorphising approach of designing AIs may be a threat to human-to-human relationships, and may thereby undermine the distinctly human-relational nature of health, that is not to say that non-humans cannot contribute significantly to general well-being. Furthermore, while animals have been useful, and some research is being done to consider robotic animals for healthcare—such as PARO, the robotic seal for dementia patients^78^—we believe that humans are capable of having numerous, intimate, personal relationships with non-humans that (in normal circumstances) do not threaten or replace human-to-human relationships. Humans have long dreamt of interacting intimately with non-human persons. For example, gods, angels, and extra-terrestrial aliens. These may, or may not, be ‘real’ beings. Considering that general artificial intelligence is predicted to be just a few years away^79,80^, it is possible that AIs, and MAIs in particular, may be the realisation of these dreams. Humans have a very high capacity for healthy relationships with both human and non-human beings (real or not). Rather than threatening or replacing DPRs, we would argue that it would be preferable to introduce complementary, yet distinctly different, healthcare relationships with xenomorphic MAIs.

## Concluding remarks: the advantages and limitations of xenomorphic MAIs

There are numerous advantages to the position proposed here. First, it avoids the challenge of the uncanny valley by removing entirely the almost insurmountable burdens of designing AIs that are indistinguishable from human beings. This burden has been the obsession of many in the field of robotics and AI and has diverted significant scientific and financial resources only to encounter the paradox whereby greater success has led to greater failure—the more human-like AIs have become, the more repulsive they appear. The xenomorphic approach avoids this paradox entirely. Second, the drive to anthropomorphise AIs is a minefield of bias. Framing AIs in human-like terms is sure to entrench existing biases that are harmful to certain social groups^81,82^. For example, research has demonstrated that people will rate humanoid AIs with long hair to be better suited for stereotypical female tasks, such as household work or care roles and less suitable for technical tasks compared with identical AIs pressed with short hair^83^. Using a xenomorphic approach grants us the opportunity to start with a clean slate and present patients with more neutral MAIs—at least as far as the presentation of these AIs is concerned. Third, with a growing body of experts raising concerns that MAIs could interfere with DPRs^84^, the xenomorphic approach may reduce this concern. While it is unlikely to entirely negate the potential conflict between human doctors and patient-facing MAIs, radically distinguishing human doctors from AIs may diminish patient confusion and help to distinguish between the different medical relationships with which patients engage.

Nevertheless, while there are clear advantages to designing MAIs to be presented in entirely alien ways, there remain limitations. First, there are questions about the suitability of interacting with an entirely alien-like AI. Would a vulnerable patient be comfortable confiding in an AI with an abnormally large head, four arms, no nose, three eyes, and scaly skin? Could such an appearance be conceived as more monster-like than alien-like? While it is true that certain forms almost instinctively elicit repulsion and fear within human beings (e.g., eight hairy legs), there is also evidence that humans can very quickly overcome this repulsion to form affectionate attachments with such creatures with the right framing. We think, for example, of popular fiction where spider-like creatures are at first perceived as horrifying but quickly become subjects of compassion. For example, the giant tarantula-like alien Hanuš in the 2024 film Spaceman, or the intelligent spider in Charlotte’s Web.

Second, as noted above, humans have an ‘exceptionally ordinary’^16^ ability to attribute human-like mental capacities to innate objects and non-human living beings^63^. Thus, it might be impossible to argue for a purely xenomorphic approach. No matter how MAIs are designed, human social cognitive abilities will ascribe to them human-like minds. This, per se, is not against our argument. There is value in a xenomorphic design for MAI, even if humans will, as they do for animals, ascribe human-like minds to these beings. Third, as further discussed above, the anthropomorphic approach presents three options, namely, A) to design a single, universally presented AI appearance, B) to present MAIs to patients according to their individual contexts, C) to allow patients to design the appearance of their own MAI. Each approach has advantages and disadvantages. The xenomorphic approach would not necessarily avoid these three options. If the xenomorphic approach were employed, it would still need to be decided which of the aforementioned approaches would be best. Should we design a single or a limited set of xenomorphic MAIs that would be presented to all patients? Or would we choose to present unique MAIs to every patient and context? For example, it may be possible to change the colour of key attributes of the MAI depending on a patient’s mood. Or would it be preferable for each person to design their own xenomorphic AI? That is to say, present patients with a range of xenomorphic features (shapes, colours, symbols, etc.). Each of these options, like the anthropomorphic approach, offers advantages and disadvantages.

Fourth, it is still possible for human beings to develop relationships with xenomorphic MAIs, which may then be used to either substitute for or replace human physician relationships. In some cases, this may be advantageous. For example, in contexts where access to a human doctor is restricted (e.g., in rural areas or in poorly performing health care systems). Yet it would not be desirable for relationships with xenomorphic MAIs to replace relationships with human physicians in contexts where human physicians are readily available (e.g., in well-performing healthcare contexts). Designing xenomorphic MAIs would not in itself preclude the displacement of human DPRs with MAI-patient relationships. However, it may make it less likely and help to preserve the value of DPRs along with their benefits.

While it is true that limitations remain to the xenomorphic approach, some of which are similar to the anthropomorphic approach, the advantages of distinctly differentiating human physicians from MAIs are significant. Reducing bias, distinguishing MAIs from human doctors, while reducing the developmental burden of MAIs should not be understated. The xenomorphic approach may help to avoid the unidirectional emotional bonds presented by anthropomorphised AIs, which present inherent dangers outlined by authors such as Schuetz^85^ by distinguishing AIs from human beings as much as possible. Yet, what is perhaps the greatest advantage is the opportunity to further develop health-affirming relationships. We have seen the benefits of adding therapy animals to healthcare contexts. It is highly probable that introducing personal, non-human, medically competent intimate relationships into healthcare could radically transform healthcare for the better.

## Data Availability

No datasets were generated or analysed during the current study.
